# Prevalence of injury and utilization of personal protective equipment among building construction workers and associated factors in Bale and West Arsi Zones, southeast Ethiopia 2022

**DOI:** 10.3389/fpubh.2024.1431797

**Published:** 2024-10-09

**Authors:** Ahmednur Adem Aliyi, Mohammed Abdela Hashim, Muhammed Jemal Abdurebi

**Affiliations:** ^1^Public Health Department, Goba Referral Hospital, Madda Walabu University, Bale Robe, Ethiopia; ^2^College of Engineering, Water Resources and Irrigation Department, Madda Walabu University, Bale Robe, Ethiopia; ^3^Department of Public Health, Institutes of Health, Bule Hora University, Bule Hora, Ethiopia

**Keywords:** construction workers, utilization, personal protective equipment, Southeast Ethiopia, injury

## Abstract

**Introduction:**

The building construction industry is well known for being one of the most dangerous industries worldwide. Statistics show it is one of the most dangerous occupations in the world. The aim of this study is to assess the prevalence of injury, personal protective equipment usage among building construction workers, and associated factors in Southeast Ethiopia.

**Methods:**

Institutional-based cross-sectional study was done among 406 selected construction workers in Southeast Ethiopia. Study participants were selected randomly. Data were collected by using a structured questionnaire. Descriptive statistics was used to summarize study variables. Binary logistic regression was utilized to investigate factors associated with injury among study participants. Accordingly, adjusted odds ratio along its 95% confidence interval were calculated and a *p* value of <0.05 was used to declare statistical significance. Generalized linear models were utilized to investigate factors associated with the use of personal protective equipment. Accordingly, an adjusted odds ratio with a 95% confidence interval was determined and a *p* value of <0.05 was used as a level to declare a significant statistical association.

**Result and discussion:**

In this research 406 building construction workers were interviewed and 393 participants gave complete responses, yielding a 96.8% response rate. Of all participants included in this study, 27 (6.8%) drink alcohol and 26 (6.6%) chew khats. Uses of personal protective equipment among study participants was 133 (33.3%, with a 95% confidence interval of 28.3% to 37.7%). Of 393 participants in this study, 213 (54.2%) of them sustained at least one building work-related injury in the last year. The commonest type of injury that occurred among this population was abrasion 43.3% followed by muscular pain (13%). Not having orientation about personal protective equipment, without safety training, didn’t use personal protective equipment, and rural residence were factors significantly associated with injury among building construction workers. Residence, age, monthly income, service year, orientation about personal protective equipment, safety training, and start using personal protective equipment immediately after the job offers were significantly associated with the uses of personal protective equipment by participants of this study. In general, this study has identified a relatively high prevalence of injury and low use of personal protective equipment in the study subjects and associated factors.

## Introduction

Occupational and industrial accidents often result from easily preventable factors that can be mitigated by implementing feasible and effective techniques, such as raising awareness among workers before they start their jobs and using proper PPE. The proper use of these procedures has led to a sustained reduction in accidents in developed countries. Therefore, the application of the abovementioned techniques can help prevent loss of life and economic costs (1).

According to the International Labor Organization and World Health Organization, approximately 2.9 million workers globally lose their lives each year due to work-related injuries and illnesses. At least 402 million workers sustain injuries at work worldwide. These organizations also stated that globally work-related illnesses cause 81% of all work-related loss of life, with death from work-related accidents resulting in the remaining 19%. They have also reported that work-related injuries and illnesses contribute to a loss of 5.4% of annual gross domestic product (GDP) growth. The other consequences due to the broader economic impact of workers’ sufferings included presenteeism (working with less effectiveness), loss of productivity because of long-term impairments, and staff turnover costs (2, 3).

Occupational injuries were most common in low-income countries, where many workers are actively working in primary and operational activities. In addition to economic losses, there are numerous costs related to significant human suffering resulting from poor occupational safety and health (OSH) conditions. This suffering is both painful and enduring, largely because it is preventable (1).

The findings from a multicenter study conducted in India showed that most workplaces are unsafe and unhealthy for the workers. These include poorly prepared work settings, unsuitable equipment, a lack of free air flow, poor lighting, sound pollution, inadequate safety in case of emergencies, and the unavailability of PPE. Workers doing their jobs in such settings are highly exposed to work-related diseases, which in turn can affect their performance and reduce productivity (4).

Personal protective equipment is essential for protecting workers from exposure to work-related hazards. Therefore, it is essential for employees to receive appropriate PPE for each potential risk and to be trained on its proper utilization. Generally, workers need a variety of equipment for different working situations. The utilization of PPE is a key measure to prevent exposure to hazards for workers in potentially dangerous environments (5). It is highly important to utilize PPE for occupational safety. It is designed to protect workers from painful disruptive effects (6).

Various studies on the use of PPE among building construction workers showed different levels of utilization across study areas (7–10).

Several studies have been conducted in Ethiopia to examine the prevalence of injuries and the use of PPE among these populations.

However, these studies were all conducted in major cities where there is close supervision and focused on the construction industry with levels one up to five only, which may affect the status of utilization of PPE (7, 11–13). Furthermore, few studies conducted on factors associated with injury and PPE use show conflicting findings (8, 9, 14–16). This indicates the need to address these factors by including a variety of building construction companies in future research. These limited and conflicting pieces of evidence report the different prevalence of injuries and PPE utilization among building construction workers and associated factors, highlighting the need for inclusive and wide-range studies. Furthermore, related studies were not conducted in the current study area. The current study assessed the prevalence of injury, PPE use, and associated factors among building construction workers in Southeast Ethiopia, encompassing a wide range of companies from various towns in the region.

## Materials and methods

### Study area, design, and period

**Figure 1:**
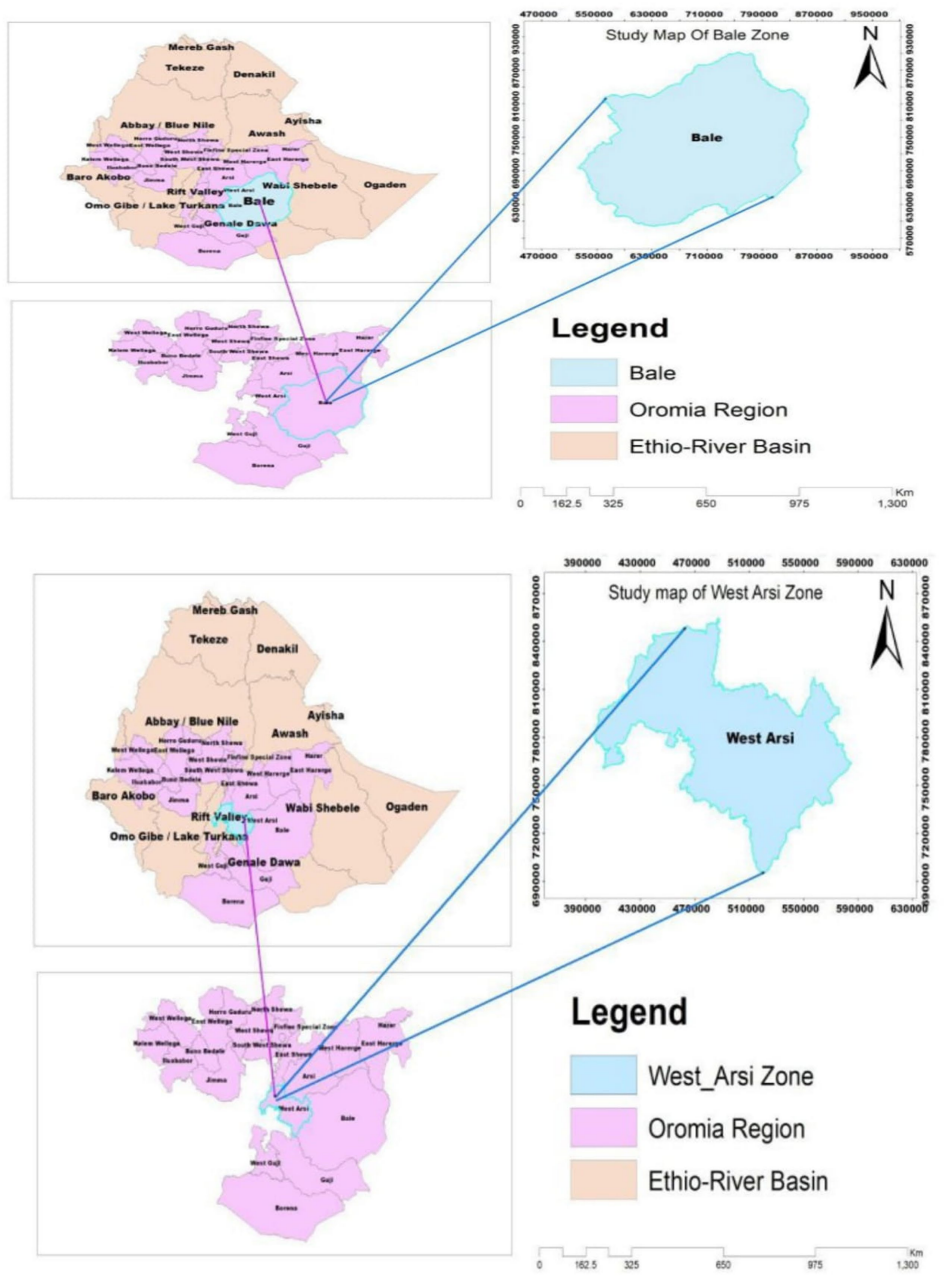
Map of the study area showing Bale and West Arsi Zones, Southeast Ethiopia.

The institutional cross-sectional study was conducted among building construction workers selected from building construction companies located in East Arsi Zone and Bale Zone, to identify the prevalence of injury, PPE use, and associated factors among building construction workers in Southeast Ethiopia from 20 October 2021 to 30 January 2022 (Figure 1).

### Population

The source population for this study included all building construction workers and those employed in management positions in building construction companies located in the Bale and West Arsi zones. Selected building construction workers available during the data collection period were considered the study population. The current study unit consisted of the selected building construction workers, including those in management positions from whom data were obtained. All building construction workers working in building construction companies in the West Arsi and Bale Zones were eligible for this study.

### Sample size and sampling procedures

To determine the required number of study participants, we used a single population proportion formula for sample size calculation. We based the calculation on an injury proportion of 39% from the same population (Fentahun (11)), with a 95% CI, a 5% precision error, and a 10% non-response rate.

The sample size was determined using the following formula:


$$n=\frac{{\left(z\propto /2\right)}^{2}\;p\;\left(1-p\right)}{d^{2}}$$

where *n* = sample size; *p* = 38.3%; *d* = marginal error = 5%.

*z* at 95% CI = 1.96.

(1.96)^2^ × (0.39) × (0.61)/(0.05)^2^.

This gave a sample size (*n*) of 365, and after adding a 10% non-response rate, the overall sample size was 406.

Regarding the sampling techniques, 60 initial lists of building construction companies with active sites were obtained from the respective zones’ construction authorities to select study participants. Next, the identified companies were grouped into two categories based on their grade. Accordingly, those with grades 1–5 and 6–10 were grouped. Twelve building construction sites (six from each group) were included using a simple random sampling based on their name list as the sampling frame. Individual construction workers were then randomly selected from the payroll list of each site.

### Data collection and quality control

Data on the prevalence of injury, PPE utilization, reasons for not utilizing PPE, and general characteristics of study participants were assessed by adapted tools developed after an extensive literature review (7, 11, 17–19). Two days of training were given to data collectors and supervisors before actual data collection.

Before data collection, the tool was pretested on 5% of the original sample size, with these subjects not included in the final study. Based on the pretest results, questions were rephrased as needed. The questionnaires were administered face-to-face by the data collectors, allowing interviewers to cross-check inconsistent answers before data cleaning.

Data completeness and consistency were checked by the data collector every time before leaving the respondent and by supervisors at the end of each data collection day. Data quality was also checked by investigators twice per week. Ethical clearance was obtained from the MWU institutional review board (Review Board Reference Number RMU-2/110/832). After providing a brief explanation of the purpose of the study to the selected companies, formal letters and written informed consent were obtained from both the companies and the study participants.

### Data processing and analysis

Sociodemographic characteristics and other general characteristics were summarized using descriptive statistics and frequency distribution. The proportion for the prevalence of injury was computed by running frequency distribution.

Furthermore, practice in utilization was identified by using a median score of practice questions and taking the median score as the cutoff point. Binary logistic regression was performed to assess factors associated with the occurrence of injury in workers. Those independent variables with a *p*-value of <0.25 in bivariate binary logistic regression were taken as candidates for multivariable logistic regression. A generalized linear model was used to assess factors associated with PPE use among building construction workers. An AOR with a 95% CI was calculated, and a p-value of less than 0.05 was used to determine statistical significance.

## Results and discussion

### Sociodemographic characteristics of study participants

In this study, 393 participants gave complete responses, yielding a 96.8% response rate. Of these participants, 302 (76.8%) were male and their mean age was 24.7 years. Based on residence, 213 (54.2%) were rural dwellers, and 340 (86.5%) were temporarily employed in their respective companies. Of the total participants, 256 (62.1%) were daily laborers in their occupation, 181 (46.1%) had attended elementary schools, and their median working time was 40 h per week (Table 1).

**Table 1:** Sociodemographic characteristics of building construction workers in Southeast Ethiopia (*n* = 393).

Variables	Category	Frequency (*n*)	Percent (%)
Gender	Male	302	76.8
	Female	91	23.2
Age in years	Mean	24.7	
	Minimum	18	
	Maximum	49	
Residence	Rural	213	54.2
	Urban	180	45.8
Employment type	Temporary	340	86.5
	Permanent	53	13.5
Marital status	Single	226	57.5
	Married	167	42.5
Ethnicity	Oromo	325	82.7
	Amhara	35	8.9
	Others	33	8.4
Religion	Muslim	179	45.5
	Orthodox	128	32.6
	Protestant	69	17.6
	Wakefata	17	4.3
Job category	Daily laborers	256	65.1
	Bar bender	35	8.9
	Plasterer	34	8.7
	Carpenter	8	2
	Masonry	17	4.3
	Electrician	18	4.6
	Site engineer	25	6.4
Income per month	Median	4,500	
	Minimum	3,000	
	Maximum	15,000	
Level of education	No formal education	33	8.4
	Elementary (1–8)	181	46.1
	High and preparatory school	144	36.6
	College and above	35	8.9
Service year	Median	4	
	Minimum	0	
	Maximum	40	
Working hours per week	Median	40	
	Minimum	14	
	Maximum	48	

### Substance use among building construction workers

**Figure 2:**
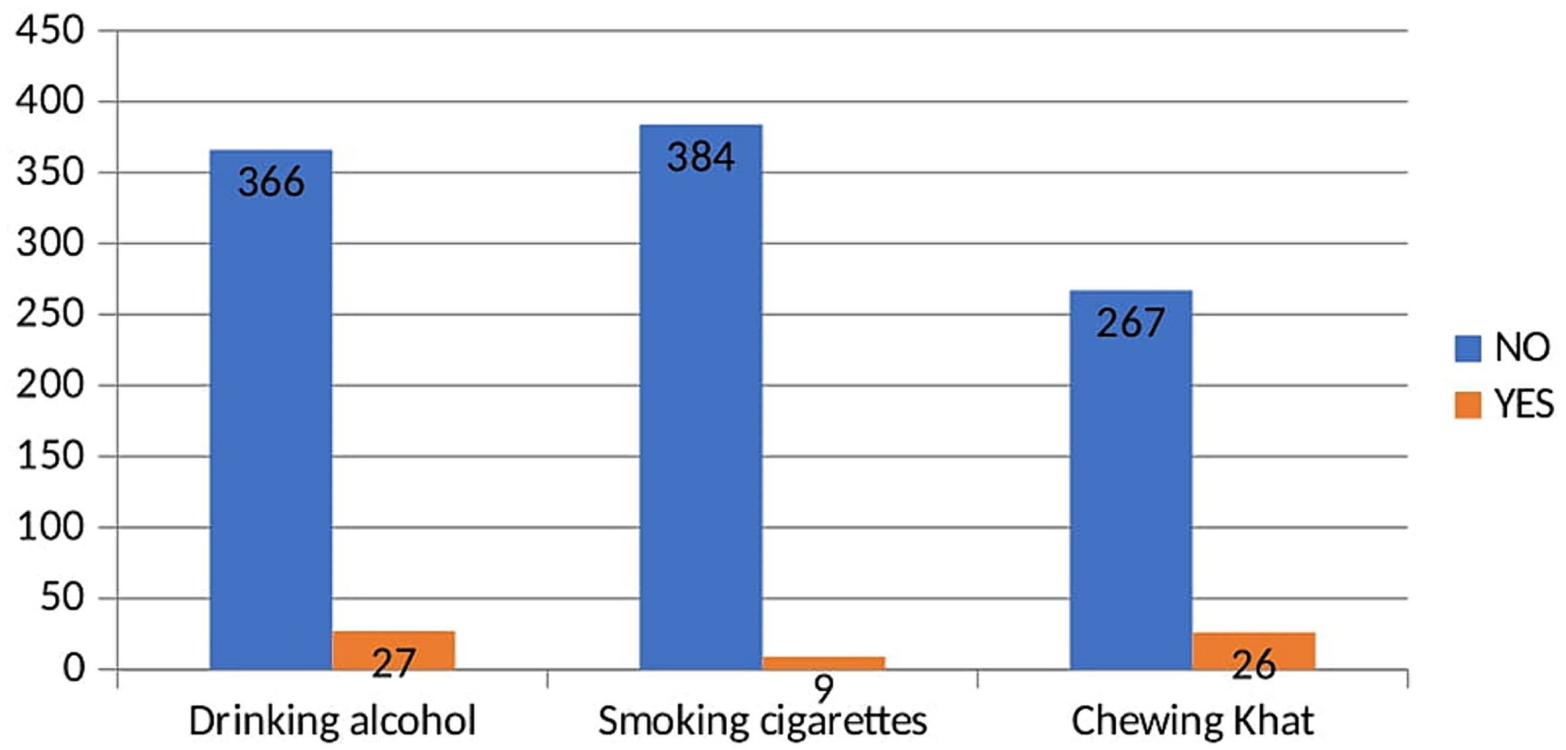
Substance use among building construction workers in Southeast Ethiopia.

Of all the participants included in this study, 27 (6.8%) drank alcohol and 26 (6.6%) chewed khats (Figure 2).

### Source of information about work-related injury

Regarding sources of information on work-related injuries, 299 (76.1%) get information from senior workers, whereas 76 (19.3%) get information from TV/radio (Table 2).

**Table 2:** Source of information about work-related injuries among building construction workers in Southeast Ethiopia (*n* = 393).

Source of information	Category	Frequency (*n*)	Percent (%)
TV/Radio	No	317	80.7
	Yes	76	19.3
Senior workers	No	94	23.9
	Yes	299	76.1
Training	No	367	93.4
	Yes	26	6.6
Poster	No	385	98
	Yes	8	2

### Prevalence and types of injury

The prevalence of building construction-related injury among workers was 213 (54.2%) with a 95% CI of 49.3 and 59.1%. The most common type of injuries among building construction workers in southeast Ethiopia were abrasion 43% (170), muscular pain 13% (51), and bone fracture 10.9% (43). Among those who were injured, 43 (91.5%) had severe injuries. The most commonly affected body part was the leg, with 53 cases (24.9%). This result was in line with a study on occupational hazards among building construction workers in southwestern Ethiopia, which reported a work-related injury prevalence of 41.4% [95% CI: (37.8, 49.4)] (17). In contrast, this finding is higher than the injury prevalence reported in studies conducted in various parts of Ethiopia and Iran, which reported rates of 38.7, 31, 41.4, 39, 38.3, and 32.6%, respectively (11, 17–21).

However, the results of the current study regarding the prevalence of building construction-related injuries were lower than those from a study conducted in Addis Ababa Ethiopia, which reported an injury prevalence of 67.7% (13). The reasons for the differences in the findings between these studies could be the time of the study, the population studied, and measurement methods. Previously studies were conducted in major cities of the country or abroad, whereas the current study included participants from outside the main cities and encompassed both complex and simple companies. The results of this study highlight the need for urgent measures to address and reduce the prevalence of work-related injuries in the building construction industry and to enhance worker safety and wellbeing (Table 3).

**Table 3:** Types of injury among building construction workers in Southeast Ethiopia (*n* = 393).

Types of injury	Category	Frequency (*n*)	Percentage (%)
Occupational injury	No	180	45.8
	Yes	213	54.2
Abrasion	No	223	56.7
	Yes	170	43.3
Muscular pain	No	342	87
	Yes	51	13
Bone fracture	No	350	89.1
	Yes	43	10.9
Strain	No	368	93.6
	Yes	25	6.4
Permanent disability	No	392	99.7
	Yes	1	0.3
Back pain	No	382	97.2
	Yes	11	2.8
Piercing	No	374	95.2
	Yes	19	4.8
Hospitalized for injury	No	166	77.9
	Yes	47	22.1
Days hospitalized due to injury	Mean	24.60	
	Maximum	45	
	Minimum	5	
	Standard deviation	8.21	
Days absent due to injury	Mean	35.06	
	Maximum	60	
	Minimum	10	
	Standard deviation	10.64	
Injury severity	Minor (absent <6 days)	0	0
	Moderate (absent 7–29 days)	4	8.5
	Severe (absent ≥ 30 days)	43	91.5
Body part affected	Hand	51	23.94
	Head	48	22.53
	Leg	53	24.9
	Axial	49	23
	Other body parts	12	5.63

### Personal protective equipment used among study participants

Of all, 133 participants (33.3%) (95% CI of 28.3–37.7%) have used at least one PPE during work. This result agrees with the findings of the research undertaken in Ethiopia (7), lower than the results of the studies conducted in Egypt and Nepal (9, 10) and greater than the results from a study conducted in Uganda (8). The reasons for these disagreements might be due to differences in the study setting, study population, and tools used (Table 4).

**Table 4:** Personal protective equipment use among building construction workers in Southeast Ethiopia (*n* = 393).

Variables	Category	Frequency (*n*)	Percentage (%)
Utilization of PPE	No	262	66.7
	Yes	131	33.3
Use hats and shoes	No	294	74.8
	Yes	99	25.2
Use gloves in rubber work	No	379	96.4
	Yes	14	3.6
Use eyepiece and facemask	No	367	93.4
	Yes	26	6.6
Use a safety belt at a height	No	333	84.7
	Yes	60	15.3
Clean and store PPE after use	No	358	91.1
	Yes	35	8.9
Wear proper closing at the workplace	No	358	91.1
	Yes	35	8.9
Wear safety boots and a mask while mixing concrete	No	376	95.7
	Yes	17	4.3
Wear safety boots during excavation	No	391	99.5
	Yes	2	0.5
Uses safety helmet	No	359	91.3
	Yes	34	8.7

### Characteristics of study participants regarding personal protective equipment

Of these populations, 298 (75.8%) had no orientation about PPE, 348 (88.5%) lacked safety training, 219 (55.7%) of their companies did not provide PPE, and 256 (65.1%) did not have PPE (Table 5).

**Table 5:** Characteristics of study participants regarding personal protective equipment in Southeast Ethiopia (*n* = 393).

Variables	Category	Frequency	Percent
Orientation about PPE	No	298	75.8
	Yes	95	24.2
Safety training	No	348	88.5
	Yes	45	11.5
Company provides PPE	No	219	55.7
	Yes	174	44.3
Have PPE	No	256	65.1
	Yes	137	34.9
Who encourages the worker to use PPE	Self	42	10.3
	PPE supervisor	35	8.9
	Safety officer	105	26.7
When did you use PPE	When start working	82	62.6
	All the time while working	46	35.1
	Only if needed	2	1.5
	When a supervisor is around	1	0.8

### Reasons for not using PPE

The common reasons for not using PPE among building construction workers were the unavailability of PPE (223, 56.7%), and the absence of strict rules in the working environment (214, 54.5%) (Table 6).

**Table 6:** Reasons for not using personal protective equipment among building construction workers in Southeast Ethiopia (*n* = 393).

Reasons	Category	Frequency (*n*)	Percent (%)
PPE not available	No	33	8.4
	Yes	223	56.7
Absence of strict rule	No	42	10.7
	Yes	214	54.5
Do not know the importance of PPEs	No	247	62.8
	Yes	9	2.3
PPE uncomfortable	No	251	63.9
	Yes	5	1.3

### Factors associated with the occurrence of injury

Binary logistic regression was used to identify factors associated with injury among building construction workers. Accordingly, bivariate binary logistic regression was performed to select candidate variables for multivariable analysis at a *p* value of <0.25. Following the results from bivariate binary logistic regression, nine variables were selected as candidates for multivariable binary logistic regression. These were the age of respondents, service year, orientation about PPE, safety training, ownership of PPE, use of PPE, employment type, gender, and residence (Table 7).

**Table 7:** Factors associated with the prevalence of injury among building construction workers in Southeast Ethiopia (*n* = 393).

Variables	Injury status		Crude OR with 95% CI	*p* value of COR	Adjusted OR with 95% CI	*p* value of AOR
	Yes	No				
Age			0.92 (0.89, 0.96)	0.001	0.96 (0.91, 1.02)	0.189
Service year			0.98 (0.95, 1.01)	0.223	0.97 (0.94, 1.01)	0.078
Have no PPE orientation	203	145	4.90 (2.35, 10.21)	0.001	3.81 (1.25, 11.59)	0.018**
Have PPE orientation	10	35	1		1	
Have no safety training	209	154	7.65 (2.59, 22.58)	0.001	5.22 (1.11, 24.65)	0.037**
Have safety training	4	23	1		1	
Do not have PPE	80	65	1.01 (071, 161)	0.167	1.95 (0.98, 3.87)	0.058
Have PPE	133	115	1		1	
Did not use PPE	204	58	47.6 (22.8, 99.6)	0.001	47 (21.27, 104.04)	0.001**
Use PPE	9	122	1		1	
Employment type						
Temporary	191	149	1.80 (1.01, 3.25)	0.048	1.66 (0.70. 3.96)	0.246
Permanent	22	31	1		1	
Gender						
Male	157	145	0.67 (0.42, 1.09)	0.110	1.24 (0.63, 2.46)	0.529
Female	56	35	1		1	
Residence						
Rural	136	77	2.36 (1.57, 3.55)	0.001	2.53 (1.35, 4.74)	0.004**
Urban	77	103	1		1	

In multivariable binary logistic regression, four variables remained significant factors associated with injury among building construction workers. These were orientation about PPE, safety training, use of PPE, and residence. Specifically, building construction workers who have not received orientation about PPE have a 3.81 times higher likelihood of experiencing work-related injury than those who have received such orientation (AOR: 3.81 [95% CI: 1.25, 11.59]). This finding was in agreement with the findings from a study conducted in Southwest and Northwest Ethiopia (11, 17). Building construction workers without safety training are (AOR: 5.22 [95% CI, 1.11, 24.65]) more likely to experience building construction-related injuries than those with safety training. This finding is consistent with the results from studies conducted in various parts of Ethiopia, Egypt, and Iran (9, 17, 19, 21–23). However, this result was different from what was identified from a study conducted in Gondar Town of Northwest Ethiopia, which reported the absence of a statistically significant association between safety training and injury among workers at that location (11).

Those who did not use PPE had a 47 times higher likelihood of sustaining injuries (AOR: 47 [95%CI: 21.27, 104.04]) than those who did. This result agrees with the results of studies conducted in various parts of Ethiopia, Uganda, Egypt, and Iran, which report a significant statistical association between not using PPE and sustaining work-related injuries (9, 12, 17–19, 21–25). This finding suggests that the use of PPE is an important predictor for preventing building construction work-related injuries.

In addition, building construction workers from rural areas are 2.53 more likely to be injured than construction workers from urban areas (AOR: 2.53 [95% CI: 1.35, 4.74]). This could be because residents of rural areas are less likely to be exposed to media informing about building construction work-related injuries and have less exposure or experience of this work compared to residents of urban areas.

The differences between current and previous studies on factors associated with construction worker injuries could be the time frame, the level of companies from which participants were selected, differences in study settings, and sample size. This means that some previous studies were conducted 5–10 years ago, with higher level building construction companies selected and in larger cities in the countries, and some studies included smaller samples than the current study.

### Factors associated with the utilization of personal protective equipment

A generalized linear model with binary logistic specification was utilized to determine factors associated with PPE use among study participants. A *p* value of <0.25 was used in bivariate analysis to identify candidate variables for multivariable analysis. Accordingly, residence, age of respondents, monthly income, years of service, orientation about PPE, safety training, company provision of PPE, immediate use of PPE after starting the job, and gender were selected as candidate variables for a multivariable generalized linear model with binary logistic regression. In multivariable analysis, all candidate variables from the bivariate analysis except gender and the provision of PPE equipment by the company remained significant factors associated with the utilization of PPE among building construction workers (Table 8).

**Table 8:** Generalized linear model with binary logistic specification results of factors affecting the utilization of PPE among study participants in Southeast Ethiopia (*n* = 393).

Variables	PPE use		Crude with 95% CI	*p* value of COR	Adjusted OR with 95% CI	*p* value of AOR
	Yes	No				
Residence						
Rural	61	152	0.63 (0.41, 0.96)	0.032	0.29 (0.16, 0.52)	0.001**
Urban	70	110	1		1	
Age in years			1.11 (1.06, 1.15)	0.001	1.14 (1.07, 1.16)	0.001**
Income per month			1.02 (1.01, 1.04)	0.001	1.01 (1.005, 1.02)	0.001**
Service year			0.98(0.94, 1.1)	0.152	0.92(0.85, 0.99)	0.034**
Have orientation about PPE	28	17	1	0.001	1	0.001**
Have no orientation about PPE	103	245	4.00 (20.4, 7.69)		6.67 (2.32, 16.67)	
Have safety training	20	7	1	0.001	1	0.006**
Have no safety training	111	255	6.67 (2.70, 16.67)		9.09 (1.92, 50.0)	
Company provides PPE	47	68	0.63 (0.39, 0.98)	0.042	1.27 (0.71, 2.28)	0.424
The company did not provide PPE	84	194	1		1	
Start using PPE immediately after the job offer	102	127	0.27 (0.17, 0.43)	0.001	0.14 (0.07, 0.3)	0.001**
Did not start using PPE immediately after the job offer	29	135	1		1	
Gender						
Male	110	192	1.91 (1.11, 3.28)	0.019	1.13 (0.59, 2.14)	0.709
Female	21	70	1		1	

Accordingly, building construction workers from rural areas are 71% less likely to use PPE than urban residents (AOR: 0.29 [95% CI: 0.16, 0.52]). This finding was not consistent with results from the study conducted in Egypt, which reported an absence of association between residence and PPE utilization among building construction workers (9). The reason for this discrepancy might be study setting and population differences. The previous study was conducted in Egypt, while the current study was in Ethiopia. As age increases by 1 year, on average, the likelihood of utilizing PPE among building construction workers increases by 14% (AOR: 1.14 [95% CI: 1.07, 1.16]). The findings from the current study regarding these factors were consistent with the findings of a study conducted in Egypt (9). However, this is not consistent with the findings of a study conducted in the selected town of Tigray Region of Ethiopia; Addis Ababa, Ethiopia; and Nepal (9, 10, 14). The reasons for these differences could be because previous studies were conducted among different populations from the current study. These studies were conducted in major cities of the selected areas, which could have frequent supervision from concerned bodies, while the current study was conducted by including building construction companies from remote towns.

As monthly income increases by one unit, the likelihood of using PPE among study participants increases by 0.1% (AOR: 1.01[95% CI: 1.005, 1.02]). This result was not consistent with the result of a study undertaken in Nepal (10), which reported no association between monthly income and PPE utilization among building construction workers. This inconsistency in the results from the current study and the earlier study might be due to differences in the study settings, as well as sociodemographic differences in the populations included in both studies.

With each yearly increment in a service year, PPE utilization decreased by 8% (AOR: 0.92 [95% CI:0.85, 0.99]). This was in line with the results of the studies from Axum and Adwa towns of the Tigray Region in Ethiopia, Nepal, and Iran (10, 14, 26). But not agree with the findings from research conducted in Addis Ababa and Egypt (7, 10). These differences could be due to the inclusion of populations from major cities in previous studies, which may result in variations in the study populations compared to the current study.

Furthermore, those who received orientation about PPE and had safety training were 6.67 and 9.09 more likely to utilize PPE (AOR: 6.67 [95% CI: 2.32, 16.67] and 9.09 [95% CI: 1.92, 50.0], respectively). These findings were consistent with results from studies conducted in different parts of Ethiopia, as well as in Uganda, Egypt, Nepal, and Iran (7–10, 21). However, they were not consistent with the findings from a study conducted in Axum and Adwa Towns of the Tigray Region, Ethiopia (14). These discrepancies could be explained due to the differences in the study population, settings, and study design used. These findings underscore the need for greater focus on improving PPE usage by addressing the identified factors that influence its utilization. The study was cross-sectional, and we recommend using a more robust study design. The study also did not address the mechanism of injury.

## Conclusion and recommendations

Current research has identified a comparatively alarming proportion of injuries and low usage of PPE among study participants. Orientation about PPE, safety training, PPE usage status, and residence were factors significantly associated with occurrences of injury among building construction workers, highlighting the need to reduce these injuries. Residence, age of workers, orientation about PPE, safety training, years of service, and immediate usage of PPE after a job offer had a significant statistical association with PPE use. Therefore, regular supervision and enforcement of PPE protocols should be consistently applied to all project contributors. Building construction workers should pay attention and prioritize their safety during all activities. In the future, researchers should conduct a study on this issue by using more robust study designs and methodologies.

## Data Availability

The original contributions presented in the study are included in the article/supplementary material, further inquiries can be directed to the corresponding author.
